# Editorial: TAVI and the Challenges Ahead

**DOI:** 10.3389/fcvm.2020.00149

**Published:** 2020-08-25

**Authors:** Ernest Spitzer, Darren Mylotte, Alexander Lauten, Crochan J. O'Sullivan

**Affiliations:** ^1^Cardiology Department, Erasmus University Rotterdam, Rotterdam, Netherlands; ^2^Cardialysis, Rotterdam, Netherlands; ^3^Cardiology Department, Galway University Hospital and National University of Ireland Galway, Galway, Ireland; ^4^Cardiology Department, Charité Medical University of Berlin, Berlin, Germany; ^5^Cardiology Department, Bon Secours Hospital, Cork, Ireland; ^6^Cardiology Department, University College Cork, Cork, Ireland

**Keywords:** transcatheter aortic valve replacement, aortic stenosis, interventional cardiolgy, prosthetic heart valve, structural heart disease, cardiac imaging

Transcatheter aortic valve implantation (TAVI) was introduced in 2002 and significant efforts in research and innovation have subsequently positioned this procedure as an important breakthrough in cardiovascular medicine (1). TAVI has become the preferred treatment strategy for symptomatic severe aortic stenosis (AS) among patients deemed to be at excessive- or high-risk for conventional surgical aortic valve replacement (SAVR) and is an alternative to surgery among intermediate- and low surgical-risk patients (2, 3).

Two decades of device iteration, refinement of procedural technique, growing operator experience, and enhanced patient selection, have significantly reduced peri-procedural complications and improved short and mid-term clinical outcomes. Central to these advances has been the concept of the Heart Team, a group of interdisciplinary healthcare professionals that each bring their experience to bear on the management of the individual patient with complex structural heart disease (1). Together the institutional Heart Team weight the anatomic, physiologic, and psychosocial aspects of each patient to develop an individualized treatment plan.

Despite the aforementioned success of TAVI, there remain important challenges to further streamline the procedure, reduce costs, and improve patient outcomes. In particular, extending TAVI to younger and lower risk patients in the aftermath of two key industry-sponsored low risk trials requires particular attention. Relevant topics for discussion include the relative merits/drawbacks of TAVI compared to SAVR, the impact and potential mitigation strategies for periprocedural stroke and permanent pacemaker implantation, the use of oral anticoagulation after TAVI, and the question of long-term transcatheter heart valve durability.

The present collection explores current and future challenges of TAVI, includes articles from a diagnostic and technical perspectives, and provides guidance on patient and vascular access selection, the importance and application of multimodal imaging, and outlines a pathway toward procedure simplification. The risk of cerebrovascular events and how to prevent them, the impact of concomitant mitral regurgitation and post-TAVI conduction abnormalities are explored and future directions in the TAVI space are discussed, including extension of the technology to younger patients, low-risk cohorts, and potentially, the role of the technology in patients with moderate aortic stenosis and heart failure.

## Pre-Procedural Planning and Technique

Advances in pre-procedural planning and refinement in procedural techniques have greatly improved clinical outcome. These developments are elegantly presented by Akodad and Lefèvre through a step-by-step approach starting with the indisputable need for a pre-TAVI multi-slice computed tomography (CT) for planning vascular access, valve type and size selection, and procedural execution. The authors describe their local experience of the benefits of fully percutaneous access, secondary radial access, conscious sedation rather than general anesthesia, rapid pacing over the left ventricular guide wire, implantation without pre-dilatation, and early discharge with limited use of the intensive care unit—restricted to patients with low-risk of post-procedural complications.

Biasco et al. dedicate a comprehensive review of vascular access sites for TAVI. The transfemoral approach is considered to be the first choice by guideline and consensus documents but alternative vascular access remains an important option in selected cases. Traditional alternate access routes include the transapical and transaortic approaches with novel and often fully percutaneous options including transaxillary, transcarotid, and transcaval accesses. The authors compare procedural success and clinical outcomes among alternate vascular access routes, underscoring that no prospective head-to-head comparisons exist.

Careful pre-procedural planning is crucial for successful TAVI. The role of MSCT imaging is particularly important in the setting of TAVI for the treatment of bicuspid aortic valve (BAV) disease. Das and Puri comprehensively summarize current data on imaging and interventional considerations in BAV. The authors highlight that asymmetric valve leaflets, the presence of a raphe, and heavy calcification among patients with BAV tend to yield more complex procedures. BAV patients undergoing TAVI may have a higher risk of valve malposition and frame underexpansion and hence increased rates of moderate to severe paravalvular regurgitation. Pre and post-dilatation can lead to aortic annular rupture if balloon sizing is excessive and the risk of ostial coronary artery occlusion and aortic dissection may be higher in patients with BAV undergoing TAVI. Recent device iteration and clinical evidence have documented improving procedural success and clinical outcomes among BAV patients, and prospective randomized controlled trials compared to surgery are called-for in this field.

O'Sullivan et al., in an original publication discuss pulmonary hypertension (PH) in patients undergoing TAVI. PH is a common finding in patients with severe symptomatic AS and has been associated with worse clinical outcomes. Among TAVI candidates, most PH is post-capillary in nature and thus associated with an increased left ventricular end-diastolic pressure. The gold standard assessment is with right heart catheterization however echocardiography provides indirect measurements of the pulmonary pressures. Pre-TAVI CT can also contain important clues that can be used to screen for PH. O'Sullivan et al. describe several potential markers, including a cut-off for the ratio between the pulmonary artery and the aortic artery diameters, which may help screening of patients undergoing TAVI.

## Current Challenges

Armijo et al. have meticulously addressed the incidence, timing, relevance, and prevention of cerebrovascular events (CVE) after TAVI. In the original PARTNER tirals, CVE were thought to be higher after TAVI compared to SAVR (ascertainment bias), current suggests stroke may be more common after SAVR. In a meta-analysis including >72,000 patients, the 30-day post-TAVI stroke rate was 3.3% (4). Importantly, the majority of these events are disabling and profoundly impacting patient quality of life. Up to 95% of procedural CVEs are ischaemic and generally are related to an embolic source, including the aortic wall, calcified aortic valves, and thrombotic material. Sub-acute CVEs are usually local thrombotic events (valve-related) or caused by atrial arrhythmia. Antithrombotic strategies as well as cerebral embolic protection devices (CEPD) have the potential to reduce the frequency and impact of these events and several ongoing randomized trials in this space are discussed by the authors.

Along these lines, Demir et al. present a rigorous review of CEPD, which were introduced to mitigate the risk of CVE during the TAVI procedure. These devices capture or deflect embolic particles away from the supra-aortic vessels and have the potential to reduce CVEs. The authors summarize the available evidence for the Claret CEPD (Boston Scientific) and for the TriGuard (Keystone Heart, Venus MedTech) and describe a range of other devices in development. The Claret device is a dual filter deployed in the brachiocephalic and left common carotid arteries, but leaving the left vertebral artery unprotected. The largest trial with CEPD (*n* = 363) to date, failed to demonstrate a significant reduction in the total new lesion volume on diffusion weighted cerebral MRI in the protected territories compared to placebo (5). However, further randomized trials of this device with clinical stroke as a powered endpoint are ongoing. The TriGuard 3 CEP is a mesh filter that is positioned across the three cerebral vessels and has been recently awarded CE-mark approval. Initial data have shown reduction of new cerebral lesions as well as lower rates of clinical CVE.

Mitral regurgitation (MR) is a common echocardiographic finding in the elderly, and moderate or severe MR affects ~20% of high-risk patients undergoing TAVI. Stähli et al. detail that moderate or severe MR is associated with worse clinical outcomes post-TAVI; however, it remains unsettled whether a direct cause-effect relationship exists. Importantly, in up to 60% of patients with MR undergoing TAVI, the severity of MR is improves at 30 days post TAVI, a finding that is hampered in patients with atrial fibrillation or severe PH. The authors describe transcatheter treatment options for residual post TAVI moderate to severe MR as well as important considerations for the timing of post-TAVI management of MR. The MitraClip (Abbott Vascular) has recently gained attention after the results of the COAPT trial which showed a significant reduction of mortality and hospitalizations in heart failure patients with moderate to severe MR, when compared with medical therapy (6).

Conduction abnormalities remain a common complication after TAVI due to the anatomical vicinity of the conduction system and the landing zone of the bioprosthetic valve. Mangieri et al. discuss in-depth the frequency and impact of new-onset left bundle branch block (LBBB) and other conduction disturbance and the need for permanent pacemaker (PPM) implantation post-TAVI. The type of device utilized, the depth of implantation, and the need of pre- and post-dilation to reduce paravalvular regurgitation are major determinants. Acute TAVI-related injury to the conduction system may cause LBBB in ~10–30% of patients and complete atrioventricular block (AVB) in ~10–30%. Importantly, LBBB may resolve in up to 85% of patients while complete AVB may resolve in up to 50%. The decision to implant a PPM therefore, should be couple with sufficient in-hospital telemetry monitoring.

## Future Directions

At the 2019 American College of Cardiology Annual Scientific Meeting, data from two large randomized controlled trials in low-risk cohorts were presented (2, 3). Balloon-expandable and self-expandable THV devices were non-inferior to SAVR for the pre-specified endpoints, and hence these trials will have far-reaching implications on current and future TAVI practice. The cohorts treated in these trials were almost 10 years younger than those included in high-risk cohorts. As De Backer and Søndergaard predicted in this section, the expansion to younger populations highlights the need for understanding the use of TAVI in patients with BAV: this morphology accounts for up to 50% of severe AS cases in patients <75 years of age. Long-term valve durability data is being collected, however, given that BAV has been largely excluded from RCTs, high quality registries or dedicated RCTs are required to further characterize the durability of TAVI prosthesis in BAV morphology. Furthermore, rates of complications may be distinctive in younger in BAV cohorts and merits careful investigation.

Voigtländer and Seiffert diligently explain this shift in focus from the early narrow high-risk TAVI cohort to the current broad all levels of surgical risk TAVI population. Patient has to date been classified according to the Society of Thoracic Surgeons Predicted Risk of Mortality (STS-PROM) at 30 days; being <4% low, 4 to 8% intermediate, and >8% high risk patients. However, this score does not include other important factors such as active malignancy, frailty, porcelain aorta, chest wall radiation, liver cirrhosis, or neurological impairment, which are important for the Heart Team decision-making. Other factors favoring TAVI include age >75, prior cardiac surgery, restricted mobility or anticipated prolonged rehabilitation, transfemoral access, severe chest deformation, or prosthesis-patient mismatch. The authors present highlights of data stemming from RCTs and registries that support the use of TAVI in intermediate-risk and data being gathered for low-risk cohorts.

Finally, Spitzer et al. open a new chapter for TAVI by providing the rationale for exploring this breakthrough therapy in patients with moderate AS in the presence of reduced left ventricular ejection fraction (LVEF). This new therapeutic target represents 0.8% of patients referred for echocardiographic assessment, and without intervention have been associated with a high rate of mortality and heart failure hospitalizations. The epidemiology, natural history, and patient characteristics are discussed, as well as the challenges in echocardiographic diagnosis of moderate AS in patient with reduced LVEF. An ongoing trial (NCT02661451) is testing the role of TAVI in this patient population. If proven successful, implementation of new clinical pathways to identify and derive these patients to a TAVI operator in a timely manner will be required, since currently patients with moderate AS are not considered a target of therapy.

## Author Contributions

All authors listed have made a substantial, direct and intellectual contribution to the work, and approved it for publication.

## Conflict of Interest

The authors declare that the research was conducted in the absence of any commercial or financial relationships that could be construed as a potential conflict of interest.
